# Changes in route of hysterectomy in Norway since introduction of robotic approach

**DOI:** 10.52054/FVVO.13.1.005

**Published:** 2021-03-31

**Authors:** ML Johanson, M Lieng

**Affiliations:** Department of Clinical and Molecular Medicine, Norwegian University of Science and Technology, 7491 Trondheim, Norway; Division of Gynecology and Obstetrics, Oslo University Hospital, 0424 Oslo, Norway; Institute of Clinical Medicine, University of Oslo, 0316 Oslo, Norway

**Keywords:** Hysterectomy, robotic hysterectomy, surgical approach hysterectomy

## Abstract

**Introduction:**

From 2008, several Norwegian Health Trusts have acquired surgical robotic systems, and robotic hysterectomy accounted for 15 % of all hysterectomies performed in Norway in 2018. Robotic assisted hysterectomy is costly, and there is no evidence that the clinical outcome of robotic assisted hysterectomy is superior compared to the outcomes following other minimal invasive hysterectomies such as vaginal and laparoscopic hysterectomies. The objectives of this study were to describe the implementation of robotic hysterectomy and changes in other hysterectomy approaches, such as open abdominal, laparoscopic and vaginal hysterectomy in hospitals with and without robotic systems for hysterectomy.

**Methods:**

Quantitative study based on hysterectomy data between 2010 to 2018 from the Norwegian Patient Registry.

**Results:**

9 out of 19 health trusts performed robotic assisted hysterectomy during the study period. The rate of abdominal hysterectomies declined during the study period, both in the health trusts with and without available surgical robotic systems. The rate of other minimally invasive hysterectomies also declined in some health trusts after the implementation of robotic assisted hysterectomy.

**Discussion:**

Robotic hysterectomy has been implemented and is increasing in Norway without a thorough evaluation of the effect on patient safety and possible economic consequences. According to our findings, it appears that the implementation of robotic hysterectomy has not had a significant impact on the use of open abdominal hysterectomy. Although associated with increased costs and a lack of evidence of improved clinical outcomes for women, robotic hysterectomy has furthermore to some extent replaced other minimal invasive hysterectomies.

## Introduction

Minimal invasive hysterectomy techniques have significant advantages, such as reduced risk of complications, shorter hospital stay and convalescence and better cosmetic result compared to open abdominal hysterectomy (Aarts et al., 2015; Billfeldt et al., 2018; Walsh et al., 2009; He et al., 2016). Around 4.800 hysterectomies are performed annually in Norway. During the period 2008 to 2018, the number of hysterectomies in Norway has remained relatively stable between 4.500 and 5.000 yearly (Johanson et al., 2020). The rate of minimal invasive hysterectomies has increased in Norway over the last two decades, and accounted for 73 % of all hysterectomies in 2018 (Goderstad et al., 2009; Johanson et al., 2020).

Minimal invasive hysterectomy traditionally included laparoscopic and vaginal hysterectomy. Lately, robotic assisted hysterectomy has been introduced as another option for minimal invasive hysterectomy. The use of robotic assisted hysterectomy is widely discussed in the literature. Several systematic reviews have found no or weak evidence to suggest that robotic assisted hysterectomy is more beneficial than other minimally invasive methods in women suffering from benign gynaecological conditions (Aarts et al., 2015; Tapper et. al., 2014; Albright et al., 2016). However the benefits of robotic assisted hysterectomy may vary by indication and individual patient characteristics, and robotics might be more beneficial in more complicated cases. In a systematic review studying robotic assisted hysterectomy in obese and morbidly obese women, robotic assisted hysterectomy was found to be a safe method (Iavazzo and Gkegkes, 2016). In addition, Moawad et al. (2017) compared robotic assisted hysterectomy with laparoscopic hysterectomy in women with larger uteri (> 750g) and found robotic hysterectomy to be a more cost effective and quicker method when the procedure was performed by surgeons who had previously performed a high number of robotic procedures.

Several studies have found robotics to be the most expensive hysterectomy method (Tapper et al., 2014; Zakhari et al., 2015; Wright et al, 2013), although this matter is widely discussed.

The first robotic assisted hysterectomy in Norway was performed in 2010. Since then, the annual number of robotic hysterectomies in Norway has increased from 26 procedures in 2010 to 713 procedures in 2018. In 2018, 15 % of the total number of hysterectomies in Norway was performed using surgical robotic systems (Johanson et al., 2020). The implementation of robotic hysterectomy in Norway, and the impact of this implementation on methods for hysterectomy has, to our knowledge, not been studied previously. The objectives of this study were to describe the implementation of robotic hysterectomy and changes in other hysterectomy approaches, such as open abdominal, laparoscopic and vaginal hysterectomy, in hospitals with and without robotic systems.

## Methods

All gynaecological hysterectomies performed in Norway from 2010-2018 was obtained from the Norwegian Patient Registry (NPR) on an individual level. In order to include all procedures, all hysterectomies according to the The NOMESCO Classification of Surgical Procedures (NCSP), except obstetric hysterectomies, were included. The following variables were obtained for each case: the woman`s year of birth, year of hysterectomy, hospital trust, diagnosis and robot assisted procedure. Procedures coded with two or more conflicting NCPS-codes were excluded from the analysis.

The data was delivered from NPR in a locked SPSS file that could be assessed with a code. The data was analysed using IBM SPSS statistics (version 26, IBM Corporation, New York, USA) and described descriptively.

The Regional Committee for Medical and Health Research Ethics concluded that the study did not need approval (REK Sør-Øst B, ref. 28752). Exemption from the law of patient confidentiality was approved by Regional Committee for Medical and Health Research Ethics September 27th, 2019 (REK Sør-Øst B, ref. 28551). Furthermore the study was approved by the Advisory Committee on the Protection of Patients Records at the Norwegian University of Science and Technology.

## Results

**Figure 1:**
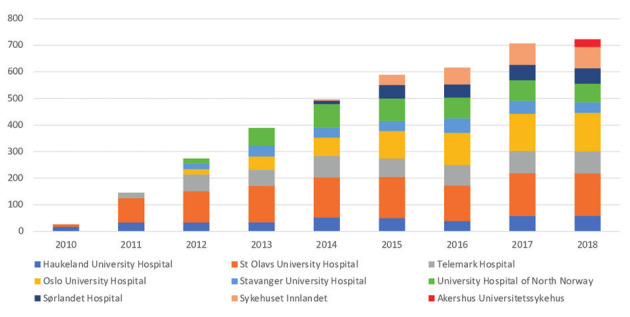
— Robotic hysterectomies in Norwegian Health Trusts 2010-2018.

In 2010, 2 out of 19 (10 %) Health Trusts in Norway reported performing robotic hysterectomies. During 2011 to 2018 this number increased to nine Health Trusts (47 %) (Figure 1). Figure 2a and b show hysterectomy approaches in hospital trusts with and without robotic systems by year. The rate of open abdominal hysterectomy declined in most health trusts during the study period, but health trusts that did not report performing robotic hysterectomy appeared to have the lowest rate of open abdominal hysterectomy, although analyses testing for statistical significance was not performed (Figure 2c). After the implementation of robotic hysterectomy, the rate of laparoscopic hysterectomy increased in six, and decreased in three health trusts (Table I). The rate of vaginal hysterectomy decreased in all health trusts following the implementation of robotic hysterectomy (Table I). Consequently, it appears that robotic hysterectomy mainly replaced open abdominal and vaginal hysterectomies in most health trusts.

**Figure 2a:**
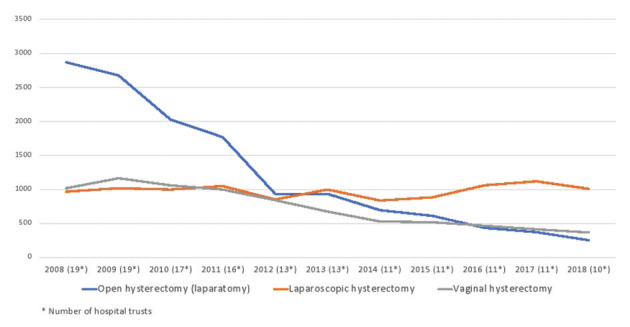
— Hysterectomy approaches in Norwegian Health Trusts not performing robotic hysterectomy 2008-2018.

**Figure 2b:**
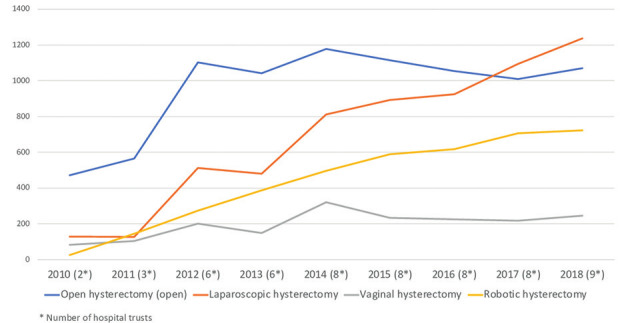
— Hysterectomy approaches in Norwegian Health Trusts performing robotic hysterectomy 2010- 2018.

**Figure 2c:**
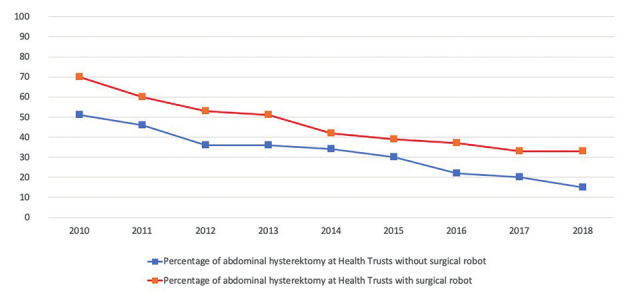
— Percentage of abdominal hysterectomies in Norwegian Health Trusts with and without surgical robot available for robotic hysterectomy 2010- 2018.

**Table I:** Distribution of hysterectomy approaches at all Norwegian Health Trusts performing robotic hysterectomy, the last year before implementation of robotic hysterectomy and 2018.

Health Trust	Distribution of hysterectomy approach the year before implementation of robot and 2018 (%)			Relative change (%)
Haukeland University hospital (2009/2018)	AH	78	42	↓ 46
	LH	13	40	↑ 208
	VH	9	2	↓ 78
	RH	-	16	-
St. Olavs University Hospital (2009/2018)	AH	59	46	↓ 22
	LH	22	2	↓ 91
	VH	19	13	↓ 32
	RH	-	40	-
Telemark Hospital (2010/2018)	AH	51	5	↓ 90
	LH	16	30	↑ 88
	VH	33	7	↓ 79
	RH	-	58	-
Stavanger University Hospital (2011/2018)	AH	62	31	↓ 50
	LH	5	38	↑ 660
	VH	33	12	↓ 64
	RH	-	20	-
Oslo University Hospital (2011/2018)	AH	48	39	↓ 19
	LH	50	45	↓ 10
	VH	2	2	-
	RH	-	14	-
University Hospital of North Norway (2011/2018)	AH	69	42	↓ 39
	LH	13	24	↑ 85
	VH	18	5	↓ 72
	RH	-	29	-
Innlandet Hospital (2013/2018)	AH	27	11	↓ 59
	LH	47	55	↑ 17
	VH	26	13	↓ 72
	RH	-	21	-
Sørlandet Hospital (2013/2018)	AH	33	26	↓ 21
	LH	39	33	↓ 15
	VH	28	17	↓ 39
	RH	-	23	-
Akershus University Hospital (2017/2018)	AH	33	22	↓ 33
	LH	46	57	↑ 24
	VH	20	10	↓ 50
	RH	-	10	-

## Discussion

Previous studies have found robotic hysterectomy to be the most expensive approach for hysterectomy, and it has not been proven that the use of robotics substantially increases quality or patient safety compared to other minimally invasive hysterectomies, such as laparoscopic and vaginal hysterectomy (Tapper et al., 2010; Lawrie et al., 2019; Wright et al., 2013; Albright et al., 2016). Nevertheless, 9 out of 19 hospital trusts in Norway perform robotic hysterectomy, and the number of hysterectomies performed with the use of the robot is increasing. Laparoscopic and vaginal hysterectomies have clinical and socioeconomic benefits compared to abdominal hysterectomy (Aarts et al., 2015; Billfeldt et al., 2018, Walsh et al., 2009, He et al., 2016). Implementing robotic hysterectomy may potentially result in a further reduction of the use of laparotomy, and could thus be defended from a quality - and patient perspective. According to our findings, implementation of robotic hysterectomy does not appear to reduce the use of the open abdominal approach significantly. However, it is likely that other factors such as increased endoscopic competence, higher obesity rate in the general population, and changed perception of aetiology and surgical treatment of gynaecological conditions have contributed to the observed changes in hysterectomy trends. Furthermore, in some hospital trusts, robotic hysterectomy appears to have replaced other minimally invasive methods, especially vaginal hysterectomy, despite lower cost and similar clinical outcomes. A part of this shift might be explained as a temporary effect, as it is likely that simple hysterectomies have been selected for the robotic approach in the learning phase during initial implementation. In our opinion, the introduction of new surgical techniques without thorough evaluation of cost and patient benefits should be avoided. In addition, robotic hysterectomies are often performed by specialised teams of gynaecologists and surgical nurses, and trainees are seldom included. Increased use of robotic hysterectomies could thus potentially result in a lower volume of procedures available for the training of new surgeons. It is well known that a lower surgical volume often results in a higher complication rate (Ruiz et al., 2018). In our opinion this effect is especially challenging in Norway, where long distances and a small population results in a high number of health trusts with small hysterectomy volumes.

This study includes a high number of hysterectomy cases, which makes it suitable for studying hysterectomy trends. All Norwegian hospitals must report all surgical cases to NPR in order to receive funding, it is therefore likely that a high proportion of the performed hysterectomies are reported. A weakness of this study is the descriptive design. We have no way of determining how the use of different surgical methods had changed if the health trusts in question had not implemented robotic hysterectomy.

We conclude that the use of robotic assisted hysterectomy is increasing in Norway, without a thorough evaluation of the effect on clinical outcomes, patient safety and possible economic consequences.
